# A review on the processing of functional proteins or peptides derived from fish by-products and their industrial applications

**DOI:** 10.1016/j.heliyon.2023.e14188

**Published:** 2023-03-01

**Authors:** Sudha Rani Ramakrishnan, Chae-Rim Jeong, Jin-Woo Park, Seung-Sik Cho, Soo-Jung Kim

**Affiliations:** a Department of Integrative Food, Bioscience, and Biotechnology, Chonnam National University, Gwangju, 61186, Republic of Korea; b Department of Pharmacy, College of Pharmacy, Mokpo National University, Muan-gun 58554, Republic of Korea; c Biomedicine, Health & Life Convergence Sciences, BK21 Four, College of Pharmacy, Mokpo National University, Muan-gun 58554, Republic of Korea

**Keywords:** Bioactivity, Byproduct, Fish, Hydrolysate, Low molecular weight, Peptide

## Abstract

To understand the production and characteristics of protein hydrolysates pertaining to individual fish species, we selected and analyzed the most important commercial fish species according to the market value based on the Statistics on International Exports of Fishery Commodities by Food and Agriculture Organization. Accordingly, salmon, shrimp, cod, tuna, squid, and herring are marine species with high global value. Peptides obtained from their by-products were predominant in hydrophobic amino acids such as alanine, phenylalanine, methionine, proline, valine, tyrosine, tryptophan, leucine, and isoleucine. Bioactive peptides are short with a length of 2–20 amino acids. They remain inactive when they are within their parent proteins. Low molecular weight (0.3–8 kDa) peptides from hydrolyzed protein are easily digestible, readily absorbed by the body and are water-soluble. The hydrophobic nature contributes to their bioactivity, which facilitates their interactions with the membrane lipid bilayers. Incomplete hydrolysis results in low yields of hydrophobic amino acids. The glycosylation type of the resulting peptide fragment determines the different applications of the hydrolysate. The degree of conservation of the glycosidic residues and the size of the peptides are influenced by the method used to generate these hydrolysates. Therefore, it is crucial to explore inexpensive novel methodologies to generate bioactive peptides. According to the current studies, a unified approach (*in silico* estimation coupled with peptidomics) can be used for the identification of novel peptides with diverse physiological and technological functions. From an industrial perspective, the reusability of immobilized enzymes and membrane separation techniques (e.g., ultrafiltration) on marine by-products can offer low operating costs and higher yield for large-scale production of bioactive peptides. This review summarizes the production processes and essential characteristics of protein hydrolysates from fish by-products and presents the advances in their application.

## 1 Introduction

By-products are edible or inedible raw materials that are leftover following the manufacture of commercial products [1]. The by-products of the fish processing industry discarded as waste vary depending on the fish species and account for almost 55%–65% of the total weight of the catch per year. This includes 15%–20% of fillet remains, 12%–18% of viscera, 9%–15% of bones, 9%–12% of heads, 5% of scales, and 1%–3% of skin and fins [2]. Although the possibilities for the commercialization of specific by-products such as intestines, bones, and blood are limited, there is ample opportunity for the development of high-value-added products. Fish by-products are known to contain functional proteins/peptides (FPPs) that can be exploited in a variety of markets and products [3].

Previous studies have prepared protein hydrolysates (PHs) from the skins of various fish species such as *Priacanthus macracanthus* and *Lutjanus vitta* [4,5]. PHs contain a wide variety of bioactive peptides with angiotensin-converting enzyme (ACE) inhibition, antimicrobial, antioxidative, and immunomodulatory properties that can be released through enzyme hydrolysis [6]. Moreover, fish skin is rich in collagen, making it ideal for the preparation of collagen hydrolysates. FPP intake promotes the regulation of the immune system and controls metabolic diseases.

Additionally, the composition and proportion of hydrophobic amino acids such as alanine, phenylalanine, proline, valine, leucine, and isoleucine in the peptide sequence determine the efficacy and bioactivity of the peptides [7]. Low-molecular-weight peptides also possess excellent biocompatibility properties, making them ideal choices for wound healing applications and medical dressings. The collagen in fish scales can be hydrolyzed to form peptide mixtures, which are effective natural inhibitors of melanin synthesis [8]. FPPs are also used in health foods and agriculture. Several brands of commercial functional foods containing oligopeptides from fish proteins such as Amizate® (The Norwegian Food Safety Authority), Stabilium® 200, and Nutripeptin® are reported to have health-promoting effects.

For obtaining the fish FPPs, the by-product is subjected to several steps such as cleaning, mincing, lyophilization, solubilization, precipitation (isoelectric point), hydrolysis (acidic, alkaline, enzymatic, or their combination), fractionation (ultrafiltration membranes or electrodialysis), and purification (size exclusion or ion exchange chromatography) [9]. We examined and abridged the available information to understand the various process parameters for the industrial production of FPPs from different fish species.

In the present study, we investigated the global market data; pre-treatment processes; properties and potentials of low-molecular-weight PHs; and applications of FPPs. These data were obtained for the by-products of marine resources with high market value such as salmon, shrimp, cod, tuna, squid, and herring. Therefore, this review provides consolidated data from the existing literature until 2022 for the development of low-molecular-weight PHs from fish by-products and to expand their application scope.

## 2 Statistics and analysis of fish species

### 2.1 Statistics of global fish production

The searches were conducted during March 2022 in the Google Scholar and PubMed database. The search result was limited to research published during the last ten years. The database search was accompanied with a manual review of the lists of relevant reference articles, which resulted in a few additional articles included in the study. Research on salmon, shrimp, cod, tuna, squid, and herring were included and studies based on the design and synthesis of bioactive peptides were excluded.

The status of global fish production was obtained from fishery and aquaculture statistics reports released by the Food and Agriculture Organization (FAO) of the United Nations [10]. The data presented in Sections 5, 6, 7, 8, 9, 10 were analyzed from over 80 references to assess the attributes of by-products of different fisheries.

### 2.2 Selection of fish species

**Fig. 1:**
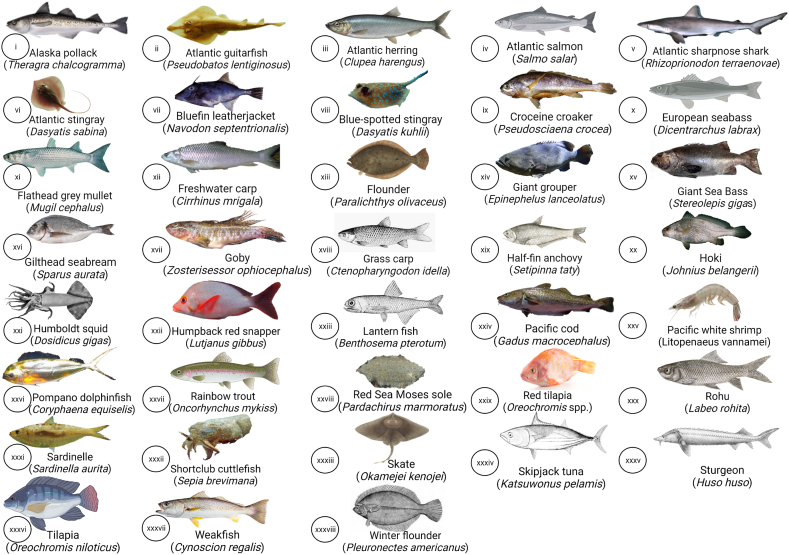
Fish species (i-xxxviii) for obtaining protein hydrolysates.

**Fig. 2:**
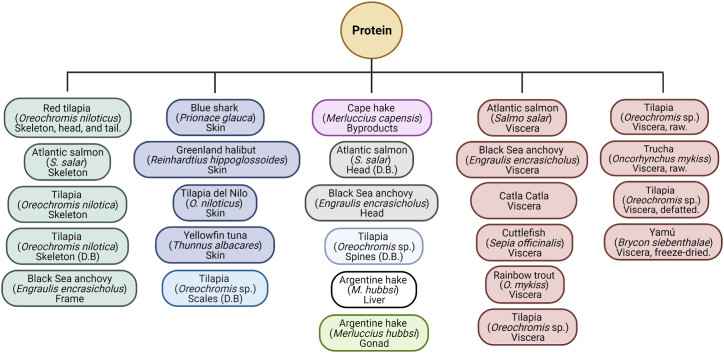
Fish species and by-product types to obtain functional proteins.

Among the available references on fish peptides, articles on processing methods of marine by-products to obtain FPP were selected for this study. The various fish species used for obtaining fish hydrolysates are summarized in Fig. 1. The fish species and their respective by-products used for obtaining FPP are provided in Fig. 2. The articles were further narrowed down to studies on one crustacean and five northeast Atlantic marine fish species, including four types of teleosts and one cephalopod, namely salmon, shrimp, cod, tuna, squid, and herring. These fish species were selected to cover the status of the most important commercial fish species according to market value based on the Statistics on International Exports of Fishery Commodities by FAO [10]. By-products derived from fish species were categorized according to the International Standard Statistical Classification of Fishery Commodities [11].

### 2.3 Analysis of methods for processing fish by-products

**Fig. 3:**
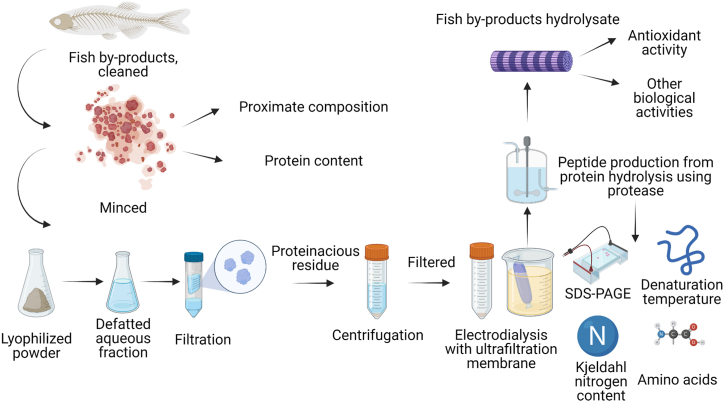
Preparation of functional protein hydrolysates.

In the present study, we analyzed the processing methods to understand the nature and dynamics of FPP prepared from by-products of fish and crustacean species. The optimum parameters for processing the by-products include the type of solvent treatment (i.e., acid, alkaline, or pressurized water), the temperature of extraction, pH for protein precipitation, and the duration of the process. The obtained protein was subjected to hydrolysis based on chemical or enzymatic processes to yield bioactive peptides of low molecular weight. Fig. 3 illustrates the overall process to obtain the hydrolysates.

## 3 Salmon by-products

### 3.1 Global market data

The global salmon market was USD 29.7 billion in 2019, with 4,926,545 tonnes being exported [10]. By-products as % of the total wet weight includes 10% head, 12.5% viscera, 10% frame, 3.5% skin, 2% trimmings, 2% blood, and 1.5% belly flap [1]. The total by-products of approximately 185 kilotonnes (kt) consist of 92 kt of head, 51 kt pf viscera, 34 kt of backbone, and 9 kt of skin [12].

### 3.2 Analysis of pre-treatment processes for industrial applications

The protein isolates are obtained as precipitates on isoelectric solubilization or pH shift [13]. It is the most common cost-effective method that can be used for a wide range of by-products and is carried out in three steps. Initially, the pH is either decreased or increased to solubilize the proteins in muscles, bones, and/or scales. Secondly, neutral lipids and disrupted cellular contents including membranes are separated by centrifugation. Finally, proteins are precipitated at their isoelectric point of pH 5.2–6. The isolated protein is subjected to acid, alkaline, or enzymatic hydrolysis to obtain the PHs. Enzymatic hydrolysis resulted in high yields of protein with improved functionalities, such as emulsification, foaming, gelling, solubility, oil holding, and water holding capacities [14].

The enzymatic protein hydrolysis (EPH) is a complex process influenced by the enzyme, by-product quality, and processing conditions, such as temperature and pH. By optimizing these factors, hydrolysates with desired bioactivity, functionality, and molecular structure can be obtained. Optimum EPH depends on the amino acid composition, degree of hydrolysis, molecular weight distribution, and product yield [15].

The activity of 3% alcalase or Flavourzyme on salmon frames for 180 min yielded 28.5–32.3 g PH/100 g sample [16]. Alcalase hydrolysates of *Salmo salar* had high surface hydrophobicity due to the presence of hydrophobic peptides resulting from the highly specific cleavage activity of the enzyme [17]. Value addition of visceral waste of Atlantic salmon (*Salmo salar*) was carried out via lactic acid (LA) fermentation or treatment with formic acid (FA) or Flavourzyme (FL, 37 °C, pH 7.0). The FL hydrolysate demonstrated high (73%) Fe (II) chelation and ferric-reducing capacity [18]. Both LA and FL hydrolysates showed enhanced antioxidant properties compared to FA hydrolysate. Anti-allergic peptides from visceral hydrolysate of Atlantic salmon (*Salmo salar*) obtained via pepsin hydrolysis (pH 2, and 37 °C) were purified and identified using Sephadex G-15 gel permeation chromatography, high performance liquid chromatography (HPLC), and LC coupled with mass spectrometry [19]. Among the fractions, C6 at 1 mg/mL exerted 89.4% anti-allergic activity *in vitro*.

Specific cationic and anionic fractions with different bioactivity responses were generated via simultaneous separation of peptides by three ultrafiltration (UF) membranes of different molecular weight exclusion limits (50, 20, and 5 kDa) stacked in an electrodialysis (ED) system [20]. This ED/UF system accelerated peptide identification in a complex salmon frame PH containing >250 different peptides.

Salmon skin PH had high level of indispensable amino acids except lysine. Salmon skin is rich in collagen, which can be used in the production of gelatin. The process includes acid or alkali treatment, pH adjustment, heating for protein extraction, and purification by filtration or ion exchange (Table 1). Fish gelatin becomes a gel at 8–10 °C, unlike bovine gelatin, which undergoes gelling at >30 °C [21]. Low gelling temperatures allow for the application of salmon gelatin in the microencapsulation of heat-labile vitamins, colorants, and flavoring agents. The debittered hydrolysate of *Salmo salar* had 36.5 g essential amino acids/100 g sample and could serve as a nutrient-rich ingredient in food fortification. Glutamine, glycine, asparagine, lysine, and leucine were the predominant amino acids in the PHs of *Salmo salar*. The size distribution varied according to the enzyme and type of by-product used for the hydrolysis [16].

**Table 1:** Properties and usage of peptides from by-products of salmon, shrimp, cod, tuna, squid, and herring.

Fish species	Recycle part	Extraction	Yield	Peptide production	Content	Sequence	Usage	Activities			Reference
								In vitro	In vivo	In silico	
Fish species	Recycle part	Extraction	Yield	Peptide production	Content	Sequence	Usage	Activities			Reference
								In vitro	In vivo	In silico	
Salmon	Head	Defatted, aqueous fraction	–	Alcalase® Ultrafiltration (0.1 μm, 1 kDa and 10 kDa)	% Protein: SHH1 (47) SHH2 (60) SHH3 (61) % Mineral: SHH (>30)	Gly-Ala-Glu-Arg-Pro, Gly-Glu-Arg-Glu-Ala-Asn-Val-Met, and Ala-Glu-Val-Gly	Nutraceuticals to prevent Alzheimer's and cardiovascular diseases	1) ORAC (μM TE/mg): SHH1 (97) SHH2 (289) SHH3 (254) 2) MCA (IC50 μg/mL): SHH1 (239) SHH2 (265) SHH3 (302) 3) ACE inhibition	–	–	[22]
	Skin	Lyophilized powder in 0.5 M acetic acid (1:10 w/v) for 72 h	–	Sample 1:20 (w/v) of 0.2% (w/v) NaCl, 0.32% (w/v) pepsin, and 0.7% (v/v) HCl, 150 rpm, 4 h, 37 °C. KH_2_PO_4_ 0.05 M. Trypsin 7% (w/v) at pH 6.8 for 6 h	16 peptides	TKLPAVF and YLNF	Functional food for prevention of type-II diabetes	Human recombinant DPP-IV enzyme inhibition IC50 (μM): TKLPVAF (242), YLNF (147)	Caco-2 cell monolayer membrane	Molecular docking permeability coefficient (3.5×10^−6^ cm s^−1^) at 5 mM, peptide allergenicity, and toxicity	[23]
	Milt	Distilled water for 15 min	7.3 g	Protease for 5 h at 50 °C, filtration through celite and combination of column chromatography techniques	12 di-, tri-, and tetra- peptides	Major peptides (dipeptides): Leu-Pro; Ile-Pro; Phe-Pro. Other peptides: Val-Pro-Ile; Ile-Pro-Ile; Ile-Pro-Leu; Phe-Pro-Val-Gly; Leu-Pro-Val-Leu; and Val-Pro-Phe-Pro	Functional food for prevention of type-II diabetes	Recombinant human DPP-IV inhibition (91%)	Postprandial hypoglycemic effect (oral starch tolerance) in Sprague Dawley rats. Clinical trial: Efficient in human subjects without insulin resistance	–	[24, 25]
	Muscle remains, head, viscera, skin, tailfins	Freeze-dried for 72 h. Pressurized liquid: Water at 1500 psi, pH 7, 50 °C, 15 min	Viscera (92%), others (28%)	Lyophilized sample (100 mg), acetonitrile (200 μL), 4 °C. Soluble peptides: 5000 rpm, 5 min	137 peptides (656.34–2601.2 Da)	Gly-Pro-Pro and Gly-Ala-Ala	Antioxidant	μM TE: TEAC (3739) and ORAC (7772)	–	–	[26]
	Frame	1 M NaOH	–	Pepsin, trypsin, chymotrypsin. 20% piperidine in dimethylformamide (v/v). Electrodialysis with ultrafiltration membrane	13 anionic and cationic peptides (456–991 Da)	IPVE, IVDI, IEGTL, LAFDHDL, LATNQHF, LVEPAAGTI, LLTEAPLN, VAPEEHPTL, LDTDYL, ILLGMD, ITDYL, IGEEF, IDAGF	Prevention of type-II diabetes	Increased glucose uptake (21%) in L6 myocytes in insulin condition at 1 ng/mL by IPVE peptide. Decreased hepatic glucose production in basal (16%) and insulin (34%) conditions by VAPEEHPTL, IVDI and IEGTL peptides. Decreased inflammation (45%)	–	–	[27]
Shrimp	Muscle, cephalothorax, shell, and tail	Freeze-dried for 24 h and powdered	–	Protease at pH 7.83 and 50 °C	7 peptides (600-1600 Da)	DEYEESGPGIVH and EQICINFCNEK	Antifreeze peptides	Ca^2+^ ATPase activity	–	Cryoprotective potential	[28]
	Cephalothorax, shells, and pleopods	By-products (300 g) in water (1:1)	–	Trypsin (45 mU/mL) at 40 °C, pH 7.1 for 2 h	<1000 Da	–	Nutraceutical	Antioxidant	–	–	[29]
	Cooking water	Filtration and freeze-drying	–	Protease (4.13 U/g) at pH 7 and 40 °C	720 Da	–	Nutraceutical	Antioxidant and ACE inhibition	–	–	[30]
	Head	No extraction. Direct hydrolysis	–	Animal protease (8.81:1 w/v, 2100 U/g, pH 7.5, 50 °C, 4.3 h)	400–550 Da	YPGE, VPW, HPLY, YATP	Glycemic control in type-II diabetes. Stability during food processing.	DPP-IV inhibition	–	Molecular docking	[31]
	Head	Degreased shrimp head and water (1:10)	–	Addition of pepsin (2500 U/g), pH 3, 4 h, 37 °C. Inactivation of pepsin at pH 8. Addition of trypsin (2500 U/g, 4 h)	180-500 Da	–	Dietary supplement for alleviation of oxidative stress and inflammation	–	Cyclophosphamide-induced hepatotoxicity	–	[32]
Cod	Blood	Centrifuge at 8000 rpm for 10 min at 20 °C	–	PW, MW and UP010 membranes at 0.1 bar	0.44 g/mL protein/peptides	–	Functional additive in food formulations, cosmetic or pharmaceutical products	Antioxidant, antimicrobial, antihypertensive	–	–	[33]
	Backbone	Homogenization	–	Hydrolysis at 300 rpm, 50 °C with 2% alcalase or Flavourzyme or 5% NaOH (w/v) for 60 min	2–15 amino acids	–	Growth promoting agents for serum-free media	Metabolic activity, proliferation, and cytotoxicity in skeletal bovine muscle cells	–	–	[34]
Tuna	Milt	Degreased and microwave pre-treatment at 420 W for 147 s	0.018–0.221 mg/kg defatted milt	Hydrolyzed with 3% neutrase at pH 7, 50 °C	GHHAAA, PHPR, AKHQ, GRVPRV, ADMYW, VDDDD, SVTEV, VKIYI, VRDQY, IRDDY, YREY, AQRPR, and SMDV	Gly-His-His-Ala-Ala-Ala, Pro-His-Pro-Arg, Ala-Lys-His-Gln, Gly-Arg-Val-Pro-Arg-Val, Ala-Asp-Met-Tyr-Trp	Protection against H_2_O_2_ damage. Natural ingredient in pharmaceutical and functional products	Antioxidant, viability of human umbilical vein endothelial cells	–	–	[35]
	Trimmings	Homogenization	–	Alcalase (3000 U/g), pH 8, 50 °C, 5 h	82% protein	ACGSDGK and KFCSGHA	Nutraceutical	Antioxidant	Antioxidant, anti-inflammation	Molecular docking with myeloperoxidase	[36]
	Muscle	Homogenization and defatted using isopropanol	–	Alcalase 2.3%, 56.2 °C, pH 9.4, 3 h	SP, VDRYF, VHGVV, YE, FEM, FWRV	Ser-Pro, Val-Asp-Arg-Tyr-Phe, Val-His-Gly-Val-Val, Tyr-Glu, Phe-Glu-Met, and Phe-Trp-Arg-Val	Functional food against hypertension and cardiovascular diseases	ACE inhibition	–	Molecular docking	[37]
	Blood	300 mL heated at 95 °C for 15 min	4.6%	Neutrase 2.9% w/w protein for 180 min at 50 °C, pH 7, and 150 rpm	Lys, Ile, Val, Leu, Phe	–	Natural antioxidants and antihypertensive peptides in food and functional products	Antioxidant and ACE inhibition	–	–	[38]
Squid	Fins	Bleached with 2% H_2_O_2_, 1:10 for 6 h at 4 °C	7.7%–11.4% (wet weight); 48.5-72.2% (dry matter)	Alcalase (30 unit/g sample)	Asp/Asn, Lys, Glu/Gln, Leu, Gly	–	Nutritional, functional, and squid flavor of various food products	Antioxidant	–	–	[39]
	Visceral ink sac	Heated to 85 °C for 10 min	–	Pepsin (0.04 U/mg), pH 3, 35 °C, 1 h	–	–	Active packaging material to maintain quality and extend shelf-life	Lipid oxidation, microbial growth	–	–	[40]
	Head	Homogenization at 11000 rpm for 1 min and autoclave at 121 °C	18.57%	Alcalase (3%), 750 min, pH 8, 50 °C	47.58 mg protein/mL	Arg-Glu-Gly-Tyr-Phe-Lys	Functional ingredient in novel food, snacks, and beverages	Antioxidant and ACE inhibition	–	–	[41]
Herring	Stickwater	Centrifugation	62.4%	Alcalase 2.4L, E/S 3%, pH 9, 55 °C, 1 h	Arg, Lys, Met, Phe, Tyr, Ile, Leu, Val, Thr, His, Glu, Gly	–	Anti-hypertension and anti-oxidation	Antioxidant and ACE inhibition	–	–	[42]
	Milt	Electrodialysis with ultrafiltration membrane	93.1%	pH 7, 6 h	–	–	Immune-metabolic disorders	–	Gut microbiota, insulin, and glucose in C57Bl/6j male mice	–	[43]
Salmon	Head	Defatted, aqueous fraction	–	Alcalase® Ultrafiltration (0.1 μm, 1 kDa and 10 kDa)	% Protein: SHH1 (47) SHH2 (60) SHH3 (61) % Mineral: SHH (>30)	Gly-Ala-Glu-Arg-Pro, Gly-Glu-Arg-Glu-Ala-Asn-Val-Met, and Ala-Glu-Val-Gly	Nutraceuticals to prevent Alzheimer's and cardiovascular diseases	1) ORAC (μM TE/mg): SHH1 (97) SHH2 (289) SHH3 (254) 2) MCA (IC50 μg/mL): SHH1 (239) SHH2 (265) SHH3 (302) 3) ACE inhibition	–	–	[22]
	Skin	Lyophilized powder in 0.5 M acetic acid (1:10 w/v) for 72 h	–	Sample 1:20 (w/v) of 0.2% (w/v) NaCl, 0.32% (w/v) pepsin, and 0.7% (v/v) HCl, 150 rpm, 4 h, 37 °C. KH_2_PO_4_ 0.05 M. Trypsin 7% (w/v) at pH 6.8 for 6 h	16 peptides	TKLPAVF and YLNF	Functional food for prevention of type-II diabetes	Human recombinant DPP-IV enzyme inhibition IC50 (μM): TKLPVAF (242), YLNF (147)	Caco-2 cell monolayer membrane	Molecular docking permeability coefficient (3.5×10^−6^ cm s^−1^) at 5 mM, peptide allergenicity, and toxicity	[23]
	Milt	Distilled water for 15 min	7.3 g	Protease for 5 h at 50 °C, filtration through celite and combination of column chromatography techniques	12 di-, tri-, and tetra- peptides	Major peptides (dipeptides): Leu-Pro; Ile-Pro; Phe-Pro. Other peptides: Val-Pro-Ile; Ile-Pro-Ile; Ile-Pro-Leu; Phe-Pro-Val-Gly; Leu-Pro-Val-Leu; and Val-Pro-Phe-Pro	Functional food for prevention of type-II diabetes	Recombinant human DPP-IV inhibition (91%)	Postprandial hypoglycemic effect (oral starch tolerance) in Sprague Dawley rats. Clinical trial: Efficient in human subjects without insulin resistance	–	[24, 25]
	Muscle remains, head, viscera, skin, tailfins	Freeze-dried for 72 h. Pressurized liquid: Water at 1500 psi, pH 7, 50 °C, 15 min	Viscera (92%), others (28%)	Lyophilized sample (100 mg), acetonitrile (200 μL), 4 °C. Soluble peptides: 5000 rpm, 5 min	137 peptides (656.34–2601.2 Da)	Gly-Pro-Pro and Gly-Ala-Ala	Antioxidant	μM TE: TEAC (3739) and ORAC (7772)	–	–	[26]
	Frame	1 M NaOH	–	Pepsin, trypsin, chymotrypsin. 20% piperidine in dimethylformamide (v/v). Electrodialysis with ultrafiltration membrane	13 anionic and cationic peptides (456–991 Da)	IPVE, IVDI, IEGTL, LAFDHDL, LATNQHF, LVEPAAGTI, LLTEAPLN, VAPEEHPTL, LDTDYL, ILLGMD, ITDYL, IGEEF, IDAGF	Prevention of type-II diabetes	Increased glucose uptake (21%) in L6 myocytes in insulin condition at 1 ng/mL by IPVE peptide. Decreased hepatic glucose production in basal (16%) and insulin (34%) conditions by VAPEEHPTL, IVDI and IEGTL peptides. Decreased inflammation (45%)	–	–	[27]
Shrimp	Muscle, cephalothorax, shell, and tail	Freeze-dried for 24 h and powdered	–	Protease at pH 7.83 and 50 °C	7 peptides (600-1600 Da)	DEYEESGPGIVH and EQICINFCNEK	Antifreeze peptides	Ca^2+^ ATPase activity	–	Cryoprotective potential	[28]
	Cephalothorax, shells, and pleopods	By-products (300 g) in water (1:1)	–	Trypsin (45 mU/mL) at 40 °C, pH 7.1 for 2 h	<1000 Da	–	Nutraceutical	Antioxidant	–	–	[29]
	Cooking water	Filtration and freeze-drying	–	Protease (4.13 U/g) at pH 7 and 40 °C	720 Da	–	Nutraceutical	Antioxidant and ACE inhibition	–	–	[30]
	Head	No extraction. Direct hydrolysis	–	Animal protease (8.81:1 w/v, 2100 U/g, pH 7.5, 50 °C, 4.3 h)	400–550 Da	YPGE, VPW, HPLY, YATP	Glycemic control in type-II diabetes. Stability during food processing.	DPP-IV inhibition	–	Molecular docking	[31]
	Head	Degreased shrimp head and water (1:10)	–	Addition of pepsin (2500 U/g), pH 3, 4 h, 37 °C. Inactivation of pepsin at pH 8. Addition of trypsin (2500 U/g, 4 h)	180–500 Da	–	Dietary supplement for alleviation of oxidative stress and inflammation	–	Cyclophosphamide-induced hepatotoxicity	–	[32]
Cod	Blood	Centrifuge at 8000 rpm for 10 min at 20 °C	–	PW, MW and UP010 membranes at 0.1 bar	0.44 g/mL protein/peptides	–	Functional additive in food formulations, cosmetic or pharmaceutical products	Antioxidant, antimicrobial, antihypertensive	–	–	[33]
	Backbone	Homogenization	–	Hydrolysis at 300 rpm, 50 °C with 2% alcalase or Flavourzyme or 5% NaOH (w/v) for 60 min	2–15 amino acids	–	Growth promoting agents for serum-free media	Metabolic activity, proliferation, and cytotoxicity in skeletal bovine muscle cells	–	–	[34]
Tuna	Milt	Degreased and microwave pre-treatment at 420 W for 147 s	0.018–0.221 mg/kg defatted milt	Hydrolyzed with 3% neutrase at pH 7, 50 °C	GHHAAA, PHPR, AKHQ, GRVPRV, ADMYW, VDDDD, SVTEV, VKIYI, VRDQY, IRDDY, YREY, AQRPR, and SMDV	Gly-His-His-Ala-Ala-Ala, Pro-His-Pro-Arg, Ala-Lys-His-Gln, Gly-Arg-Val-Pro-Arg-Val, Ala-Asp-Met-Tyr-Trp	Protection against H_2_O_2_ damage. Natural ingredient in pharmaceutical and functional products	Antioxidant, viability of human umbilical vein endothelial cells	–	–	[35]
	Trimmings	Homogenization	–	Alcalase (3000 U/g), pH 8, 50 °C, 5 h	82% protein	ACGSDGK and KFCSGHA	Nutraceutical	Antioxidant	Antioxidant, anti-inflammation	Molecular docking with myeloperoxidase	[36]
	Muscle	Homogenization and defatted using isopropanol	–	Alcalase 2.3%, 56.2 °C, pH 9.4, 3 h	SP, VDRYF, VHGVV, YE, FEM, FWRV	Ser-Pro, Val-Asp-Arg-Tyr-Phe, Val-His-Gly-Val-Val, Tyr-Glu, Phe-Glu-Met, and Phe-Trp-Arg-Val	Functional food against hypertension and cardiovascular diseases	ACE inhibition	–	Molecular docking	[37]
	Blood	300 mL heated at 95 °C for 15 min	4.6%	Neutrase 2.9% w/w protein for 180 min at 50 °C, pH 7, and 150 rpm	Lys, Ile, Val, Leu, Phe	–	Natural antioxidants and antihypertensive peptides in food and functional products	Antioxidant and ACE inhibition	–	–	[38]
Squid	Fins	Bleached with 2% H_2_O_2_, 1:10 for 6 h at 4 °C	7.7%–11.4% (wet weight); 48.5-72.2% (dry matter)	Alcalase (30 unit/g sample)	Asp/Asn, Lys, Glu/Gln, Leu, Gly	–	Nutritional, functional, and squid flavor of various food products	Antioxidant	–	–	[39]
	Visceral ink sac	Heated to 85 °C for 10 min	–	Pepsin (0.04 U/mg), pH 3, 35 °C, 1 h	–	–	Active packaging material to maintain quality and extend shelf-life	Lipid oxidation, microbial growth	–	–	[40]
	Head	Homogenization at 11000 rpm for 1 min and autoclave at 121 °C	18.57%	Alcalase (3%), 750 min, pH 8, 50 °C	47.58 mg protein/mL	Arg-Glu-Gly-Tyr-Phe-Lys	Functional ingredient in novel food, snacks, and beverages	Antioxidant and ACE inhibition	–	–	[41]
Herring	Stickwater	Centrifugation	62.4%	Alcalase 2.4L, E/S 3%, pH 9, 55 °C, 1 h	Arg, Lys, Met, Phe, Tyr, Ile, Leu, Val, Thr, His, Glu, Gly	–	Anti-hypertension and anti-oxidation	Antioxidant and ACE inhibition	–	–	[42]
	Milt	Electrodialysis with ultrafiltration membrane	93.1%	pH 7, 6 h	–	–	Immune-metabolic disorders	–	Gut microbiota, insulin, and glucose in C57Bl/6j male mice	–	[43]

SHH: Salmon head hydrolysate; ORAC: Oxygen radical absorbance capacity; TEAC: Trolox equivalent antioxidant capacity; MCA: Metal chelation activity; ACE: Angiotensin-I converting enzyme; DPP-IV: Dipeptidyl peptidase IV; IC_50_: Half maximal inhibitory concentration.

### 3.3 Applications of functional proteins/peptides

The quantity of by-products increases with the production size, and therefore sustainable use of by-products is essential. Salmon by-products are of high value, as they exhibit *in vitro* antidiabetic potential, modulate gastrointestinal stress in patients with irritable bowel syndrome, and can be included as an emollient in cosmetic formulations [44,45,46].

Atlantic salmon hydrolysate showed higher water holding capacity than egg albumin and soy protein concentrate [47], indicating its suitability for use as meat extenders. Skin collagen hydrolysates exhibited antioxidant (<3 kDa) and anti-freezing (>3 kDa) activities [48] and can thus be used as cryoprotectants in protein storage. Antioxidative peptides identified include hydrophobic amino acid residues valine or leucine at the N-terminus end and proline, histidine, or tyrosine in the sequences. The gelatin hydrolysate obtained after treatment with alcalase and Flavourzyme increased the insulin secretion capacity of BRIN-BD11 cells and showed superior dipeptidyl peptidase (DPP)-IV inhibitory activity compared to a gelatin hydrolysate obtained via alcalase and Promod™ treatment.

Two peptide fractions from salmon frame decreased LPS-induced inflammation in macrophages at 1 μg/mL (45% A_FFC_ and 30% C_FFC2_) [20]. The A_FFC_ fraction can be considered a novel natural therapeutic agent as it exhibited the same effect as phenformin at 10 μM (40%), a drug used to treat type-2 diabetes. Cationic peptides present in the A_FFC_ fraction were responsible for the anti-inflammatory properties and bioactivity of the peptides, which presumably resulted from the synergistic interaction between different peptides. Anti-allergic peptides from visceral hydrolysate of Atlantic salmon (*Salmo salar*) could be used as an alternative to hormone therapy for allergy treatment [19].

## 4 Shrimp by-products

### 4.1 Global market data

The global shrimp market was valued at USD 25.6 billion in 2019 for an estimated export of 4,678,558 tonnes [10]. The total by-products (45%–48%) include 38.9% head, 48.1% muscle, 10.7% shell, and 2.3% tail [49].

### 4.2 Analysis of pre-treatment processes for industrial applications

By-products such as cephalothorax, exoskeleton, shell, pleopods, tail, and cooking wastewater contain 45% of total proteins [50] and are subjected to deproteination for the extraction of protein, followed by enzymatic hydrolysis using proteolytic enzymes to obtain the PH. The PH obtained through chemical extraction exhibits a high degree of hydrolysis (DH) and low recovery compared to enzymatic methods [51]. An optimized aqueous two-phase partitioning system resulted in the recovery of 74.5% protein while maintaining the biological activity and solubility of the extracted proteins [52].

The optimized conditions to obtain shrimp shell waste hydrolysates with bioactivity were 5.4% (w/w) neutrase concentration, 13 mL/g liquid-solid ratio, 4.1 h hydrolysis time, 50 °C, and pH 7 [53]. The hydrolysates consisted mainly of small peptides with molecular weights less than 4 kDa and high essential amino acids (278 mg/g), which indicated its high nutritional value based on the FAO/WHO recommended standard protein. Multiple methods, namely centrifugation, microfiltration, precipitation, UF, and their combinations, are used to recover myofibrillar and sarcoplasmic proteins from shrimp waste [54]. Shrimp wastes are also processed using drying and grinding, hydrothermal carbonization, and calcification in air (to produce CaCO_3_ and CaO active compounds).

### 4.3 Applications of functional proteins/peptides

The hydrolysates of shell wastes from *Fenneropenaeus chinensis* exhibited 2,2-diphenyl-1-picrylhydrazyl (DPPH) scavenging, iron (III) reduction, and lipid peroxidation activities. Peptide fractions <3 kDa, 3–5 kDa, and 5–10 kDa from cephalothorax and abdominal parts of *Parapenaeus longirostris* as well as low molecular weight pentapeptides obtained by thermolysin hydrolysis from bycatch of *Oratosquilla woodmasoni* waste exhibited improved *in vitro* ACE inhibition and antioxidant activity [55,56]. Hydrophobic amino acids, particularly with aliphatic chains, like glycine, isoleucine, leucine, and valine are typical of the N-terminus of peptidyl ACE inhibitors. In addition, the PH having ACE inhibitory activity contained proline or aromatic amino acid at the C-terminal end of the peptide.

Carotenoproteins from shell waste of *Metapenaeus affinis*, *Nematopalemon tenuipes*, *Parapenaeopsis stylifera*, and *Penaeus monodon*, extracted using papain enzyme serves as an effective antioxidant. Superiority of carotenoprotein extracted from the shell waste of *Parapenaeopsis stylifera* has been proven to be composed of high quantity of essential amino acids, carotenoids, protein content, and enhanced antioxidant activity compared to other species [57]. The carotenoprotein can be used as a feed ingredient, nutraceutical, and color enhancer.

Low molecular weight peptides from red shrimp (*Solenocera crassicornis*) heads can be used to treat cyclophosphamide-induced hepatotoxicity [32]. Shrimp-derived peptides QMDDQ (Gln-Met-Asp-Asp-Gln) and KMDDQ (Lys-Met-Asp-Asp-Gln) exhibited neuroprotective effects by increasing acetylcholine and inhibiting acetylcholinesterase (AChE) in PC12 cells [58]. The peptide QMDDQ was more active than KMDDQ as it exhibited an extended spatial conformation, facilitating its interactions with AChE and exhibiting memory protection properties in mice.

Shrimp (*Litopenaeus setiferus*) shell was hydrolyzed using food-grade cryotin enzyme to obtain gastrointestinal resistant peptide hydrolysate [59]. The hydrolysates also possessed α-amylase inhibitory activity, the ability to scavenge 2,2-diphenyl-1-picrylhydrazyl free radicals, reduced the Fe^3+^ ions, and inhibited lipid peroxidation [53].

Shrimp is produced in large quantities and large amounts of by-products are discarded every year. Therefore, utilizing the by-products of the shrimp industry should be prioritized. Shrimp-derived peptides improved memory ability in mice and exhibited neuroprotective effects due to their N-terminal glutamine, which facilitates their interactions with acetylcholinesterase [58]. These novel neuroprotective peptides are of high value due to their low toxicity and can be developed as a potential neuroprotective peptide drug.

The FPPs from shrimp shell waste also have diverse applications such as sorbent to detect the residue of veterinary drug, radioactive material removal, benzene destruction, oil spill dispersant, and corrosion inhibitor for carbon steel.

## 5 Cod by-products

### 5.1 Global market data

The global cod market was valued at USD 16.2 billion in 2019, with an export of 9,011,626 tonnes [10]. Cod fillets represent 43% of the total fish composition. Other portions include 24% head, 12% backbone, 6% cut-offs, 5% viscera, 5% liver, 3% skin, and 2% roe and milt [60]. Approximately 4000 metric tonnes (MT) of skin yields 400 MT of collagen annually, for which almost 130,000 MT of cod is required [61].

### 5.2 Analysis of pre-treatment processes for industrial applications

Steam explosion-assisted extraction of protein from backbones improved the flavor, whereas Flavourzyme yielded high amino acid levels (151.50 mg/100 mL) without umami taste. The extracellular protease MCP-01 from deep-sea *Pseudoalteromonas* sp. SM9913 is a serine collagenolytic protease used for the efficient hydrolysis of cod skin collagen [62]. The use of high enzyme doses is not cost-effective because a large amount of hydrolysate is produced in a short time, which leads to enzyme and substrate inhibition, thus reducing the reaction rate. Decalcification with 0.5 M EDTA-2Na is one of the processes involved in the isolation of collagen from bones and scales [63]. The hydrolysates had 84.88 g protein and 70–82 hydroxyproline residues that contributed to the thermal stability of collagen [64]. The codfish skin hydrolysates from laboratory-, pilot-, and plant-scales had similar quality, containing ∼95% peptides with molecular weights lower than 3000 Da and ∼60% lower than 1000 Da, in which collagen oligopeptides account for ∼95% [62].

### 5.3 Applications of functional proteins/peptides

Matrix metalloproteinase (MMP-1) induces dermal collagen degradation. Two peptides isolated from cod skin gelatin hydrolysates inhibited phosphorylated extracellular signal-regulated kinase (p-ERK), p-p38, and MMP-1 expression at 0.1 mg/mL in fibroblasts irradiated with 20 mJ/cm^2^ of UVB, thereby arresting collagen degradation. The peptides also inhibited phosphor-c-Jun N-terminal kinase (p-JNK) in the mitogen-activated protein kinase (MAPK) signaling pathway, which is involved in the photoaging process. Therefore, these peptides could be used as a functional ingredient in the development of skin protection products [65]. Purification of papain hydrolysate yielded peptides [Thr-Cys-Ser-Pro (388 Da) and Thr-Gly-Gly-Gly-Asn-Val (485.5 Da)] with high antioxidant activity (81%) at 500 μg/mL [66], while GASSGMPG and LAYA exhibited antihypertensive activity at IC_50_ = 6.9 and 14.5 μM, respectively [67]. These peptides can be used as functional ingredients for improving cardiovascular health. Isolated peptides exert antioxidant activity due to the hydrophobic nature of some of the amino acids present in the protein hydrolysate. The mechanism of action is that the antioxidant peptides could smoothly enter the target organs through hydrophobic interactions with the membrane lipid bilayers by aid of their hydrophobic nature, where they are able to exert significant capacity of scavenging radicals. Blood processed using a UP010 UF membrane yielded bioactive peptides (10 kDa) capable of inhibiting the growth of *Escherichia coli* with high 2,2′-azino-bis(3-ethylbenzothiazoline-6-sulfonic acid) (ABTS^+^) and ORAC activities [33], which can be exploited as new alternative antimicrobial agents.

Large quantities of protein-rich by-products are generated by the fish processing industry. These by-products can be used in the development of new bio-based ingredients. Peptides from cod by-products can be used to prevent hypertension and its related diseases. Moreover, peptide powder made of collagen from cod skin is widely used in pharmaceuticals, cosmetics, and food supplements.

## 6 Tuna by-products

### 6.1 Global market data

The global tuna market was valued at USD 14.7 billion in 2019, with an export of 5,845,322 tonnes [10]. Tuna meat is 62% of the total fish composition. Other components include the head (13%), viscera (8%), bones (6%), fins (1%), and skin (10%). The non-edible (3%–5%) by-products include the operculum (gill cover) and gills [68].

### 6.2 Analysis of pre-treatment processes for industrial applications

Umamizyme is used in the production of hydrolysates from tuna stomach proteins. A DH of up to 22.5% was obtained with an enzyme/substrate (E/S) ratio of 1.5% after 4 h of hydrolysis at 45 °C and a pH of 7 [69]. However, Umamizyme is less stable than Alcalase® 2.4L. The protein concentrates produced with acetic acid contained 5% moisture, 1.2% lipids, and 89.5% protein with functional properties [70]. During storage for three months in 300-gauge polyester/polythene laminated pouches at ambient temperature, the product did not undergo browning. Treatment with 0.225 M of acetic acid for 2.5 h maximized hydration and gelatin extraction (20%) from Yellowfin tuna (*Thunnus albacares*) skin, which is an efficient source of type-B gelatin [71].

### 6.3 Applications of functional proteins/peptides

Molecules possessing structural and/or functional characteristics of gastrin and chole-cytokinin, which are secretagogue molecules exhibiting a large spectrum of activities ranging from the stimulation of protein synthesis to the secretion of digestive enzymes or cellular growth factors, have been detected in tuna stomach hydrolysates prepared using Alcalase® 2.4L. These hydrolysates can be used as nitrogenous substrates to stimulate the growth of microorganisms in the fermentation industry [72].

Short and medium-sized peptides isolated from tryptic digests of tuna myofibrillar fraction showed antimicrobial activity [73]. Anti-bacterial peptides are positively charged, whereas anti-fungal peptides display hydrophobicity. The structure of the peptide GILTLK has a hydrophobic portion composed of isoleucine and two leucine, both of which are aliphatic amino acids. The hydrophobic portion is involved in the formation of pores in the bacterial cell wall and cytoplasmic membrane, the release of cytoplasmic contents, and the destruction of the pathogen, which is a non-receptor mediated activity. Treatment at 54 °C maximized the gelling temperature, melting temperature, and gel strength [71]. This may be beneficial in formulations to achieve high storage stability of gelatin-based confectioneries, nutraceuticals, and pharmaceuticals.

Tuna hydrolysates, particularly from the head, are the best source of prolyl oligopeptidase and ACE inhibiting molecules, which could be used in the prevention and treatment of cardiovascular and neurological diseases [74]. Therefore, tuna by-products are a good source of functional foods or pharmaceuticals. Hydrolysates of tuna by-products can be used to enhance whipping, gelling, and textural properties.

## 7 Squid by-products

### 7.1 Global market data

The global squid market is valued at USD 11.3 billion, with exports of 2,686,315 tonnes [10]. Processing produces by-products (52%) including 25% head (eyes, mouth, beak) and tentacles (arms), 15% fins, 8% viscera (ink and hepatopancreas), 3% skin, 1% cartilage/pens, and 48% unclaimed mantle including the tunic [75].

### 7.2 Analysis of pre-treatment processes for industrial applications

The conversion of collagen to gelatin depends on the pre-treatment conditions, processing parameters, and preservation method of raw materials [76]. The inner and outer tunics of squid were hydrolyzed with pepsin, followed by gelatin extraction (G1) using a mild acid and second extraction (G2) of the collagenous residues. The G1 had a good gel-forming ability, whereas G2 displayed weak viscoelastic behavior and gel strength. Both G1 and G2 showed an absence of color, opacity, low water vapor permeability, high puncture deformation, and good filmogenic ability [77]. The optimal enzyme and conditions for gelatin hydrolysis included alkaline protease treatment at 7000 U/g (pH 6, 55 °C) for 109 min [78]. By-products such as arms and fins from the industrial processing of squid were utilized to extract collagen, which was hydrolyzed using protease XIV and UF to obtain the peptide fractions [79].

### 7.3 Applications of functional proteins/peptides

Value-added compounds obtained from squid by-products include protein concentrates from fins [80], proteases from hepatopancreas [81], collagen, gelatin, and bioactive compounds from the head, arms, tentacles, ink, and skin [82,83,84], bio-plasticizers, food additives, and fish meal from the head, tentacles, skin, mouth, arms, pens, and viscera [85,86,87], chitin from cartilage and pens [88], eicosapentaenoic and docosahexaenoic acids from the digestive gland, testis, and integument [89], and pigments from the skin as preservative agents [90].

The DPPH radical scavenging activity of gelatin peptide was 93.18% [78]. The UF process increased the antioxidant and antimutagenic activities of the peptide fraction (F3) <5 kDa but did not improve its antiproliferative activity. The F3 bioactivity is associated with the composition of antioxidant amino acids such as hydroxyproline, glycine, arginine, and lysine [79]. PHs of squid heads obtained using Alcalase® and Flavourzyme® showed DPPH (64.20%) and ABTS (102.50 μgmL^−1^) scavenging activities. Purification revealed that the most potent sequence was Arg-Glu-Gly-Tyr-Phe-Lys [41]. This peptide from dried squid heads can be used as an antioxidant in food.

The Maillard reaction enhanced the antioxidant (free radical scavenging activity, ferrous chelating capacity, and reducing power) and antibacterial activities of squid by-product hydrolytic peptides. The antibacterial activity of Maillard reaction products lasted longer than ampicillin, reaching >30 days [91]. Jumbo squid pens possess antibacterial activity and are therefore considered a promising low-cost alternative to conventional antibiotics. Additionally, alkali- or acid-treated by-products retain good biological activity and could thus be used as natural antimicrobial peptides in the food industry.

## 8 Herring by-products

### 8.1 Global market data

The herring market size was valued at USD 4.3 billion in 2019, with an export of 3,591,715 tonnes [10]. The total by-products (47–54%) include 16% head, 13–20% viscera, 8% skin, and 10% spine and bones [92].

### 8.2 Analysis of pre-treatment processes for industrial applications

The hydrolysis process to obtain functional proteins or peptides from the by-products includes grinding, heating with scraped surface heat exchangers, enzymatic digestion, concentration with vacuum evaporation, and pH adjustment for spoilage control. The hydrolysis time determines the amount of tryptophan (essential amino acid) available in the protein hydrolysate [93]. Hydrolysates are easily digestible (related to the solubility of the protein) and can be spray-dried for longer storage.

Pacific thread herring (*Opisthonema libertate*) muscle was hydrolyzed with alcalase. Hydrolysis using exogenous commercial enzymes obtained from plants (bromelain, papain), animals, or microbial sources is highly selective, specific with mild reaction conditions, and better control, as well as improved functionality due to the effective action of the enzymes [94]. The DH ranged between 9.6% and 33.1%. The highest DH was obtained with a 3% enzyme concentration at 50 °C and a pH of 9 [95].

Milt hydrolysate was separated into cationic and anionic peptides by ED/UF, as they display different bioactivities [96]. Odorant compounds were removed via deaerator treatment of the hydrolysate at pH 7 and alkalization to pH 10 [97]. Uncontrolled pre-treatment process can result in poor hydrolysis. This may be evident from the harsh unspecific cleaving of the peptide bond; partial or complete destruction of the amino acids namely, cysteine, serine, and threonine; as well as the formation of toxic substances, such as lysinoalanine, ornithinoalanine, and lanthionine on alkaline hydrolysis.

### 8.3 Applications of functional proteins/peptides

Ensilaging of herring by-products for 7 days at 7 °C revealed an increased rate of peroxide value, 2-thiobarbituric acid reactive substances (TBARS), malondialdehyde (MDA), 4-hydroxy-hexenal (HHE), and 2,4-heptadienal [98]. Natural antioxidant peptides with a molecular weight of <3500 Da were produced from Pacific herring protein [99]. Two cationic peptides (IVPAS and FDKPVSPLL) from milt hydrolysate decreased the inducible nitric oxide synthase (iNOS) activation at 1 ng/mL (−11.65% and −13.22%) and 100 pg/mL (−17.58% and 15.51%), demonstrating their *in vitro* anti-inflammatory effect in macrophage cells [100].

However, high antioxidant activity evaluated based on the scavenging of DPPH (183.7 μmol TE/mg), Ferric Reducing Antioxidant Power (FRAP, 0.98 μmol TE/mg), and ABTS (144.9 μmol TE/mg) was obtained at a pH of 8. The hydrolysates also exhibited a high percentage of peptides with molecular weight <1.35 kDa and high concentrations of anionic and cationic amino acids with a potential for application in functional food formulations [95]. Milt hydrolysate is used in the production of fertilizers and organic acids, such as acetic and lactic acids [97].

The search for bioactive peptides has increased over the past decade and marine organisms revealed a high richness of these peptides. Herring milt hydrolysates demonstrated *in vitro* anti-inflammatory activity, thus highlighting their potential use in the prevention of metabolic syndrome. Nevertheless, additional studies are needed to assess the toxicity and related functionalities of these peptides.

## 9 Conclusion

From our analysis of the Statistics on International Exports of Fishery Commodities, we found that among the marine species, salmon, shrimp, cod, tuna, squid, and herring are leading the global market in terms of production and export value. Thus, the quantity of byproducts obtained from these fishes are high compared to their counterparts. By-products are parts of the fish that are removed prior to reaching the end consumer to improve the shelf-life, reduce the transportation weight, and/or increase the value of the main fish product. Alkaline solubilization affects the amino acid composition, the secondary structure of the proteins, and the solubility of the fish protein isolates (FPI). The emulsifying ability depends on the concentration of FPI and the fish species and not on the conditions for obtaining the isolates. FPIs obtained from salmon, cod, and herring had low glycine. Fish waste hydrolysates contain beneficial components that can be converted to useful and potentially high-value products. Fish gelatins are preferred for low-temperature gelling applications. Short peptides containing 2–10 amino acids possess a higher antioxidant capacity and biological activity than other counterparts. Salmon by-product-derived peptides are useful as meat extenders, antioxidants, cryoprotectants, and functional materials for the treatment/prevention of type-2 diabetes. Shrimp by-products can be utilized as ACE-inhibitors, antioxidants, feed ingredients, nutraceuticals, color enhancers, hepatotoxicity alleviators, and neuroprotectors. Cod skin gelatin hydrolysates are non-toxic and non-irritant to the skin due to their moisture-retention ability, in addition to promoting the viability of human dermal fibroblasts and possessing antioxidant and antihypertensive properties. Tuna hydrolysates can be used as microbial growth substrates, food texture enhancers, and for cardiovascular protection. Protein concentrates, proteases, bio-plasticizers, and preservative agents can also be obtained from squid by-products. Herring by-products can be utilized as anti-inflammatory and antioxidative agents. The different applications of the hydrolysates can be attributed to the glycosylation types in the resulting peptide fragments. The method used to generate these hydrolysates may also influence the degree of conservation of the glycosidic residues. Sensorial properties of FPPs are dependent on the fish species. For this reason, despite, hydrolysates from fish by-products have the potential to be used as food ingredients, the application varies with the fish species.

## 10 Future directions and development strategies for high value proteins/peptides derived from fish by-products

The application of peptides in functional and sports foods or drinks could be an interesting option if the hydrolysis of by-products renders a soluble tasteless protein. Research focused on identifying useful properties of fish proteins could enable the replacement of traditional sources in ways such as (1) to prevent the use of potentially dangerous peptides (e.g., those derived from the bovine spongiform encephalopathy crisis) or (2) to provide alternatives to porcine gelatin among people with religious restrictions. Fish skin is a good source of gelatin. The global market for gelatin is extremely large, as it is used in a wide variety of products, ranging from puddings to gummy bears in the food industry, as well as face masks and capsules in the pharmaceutical industry. Additionally, the market for fish gelatin is steadily growing due to the increasing consumer demand for products free of farmed animals. Valorization of discarded biomass leads to profitable products for different sectors such as the food, agrochemical, medical, and pharmaceutical industries. These activities ensure the sustainability of fish resources and novel technologies could contribute to the effective utilization of fish processing waste. The huge global supply of fish by-products could serve as a low-cost source of proteins and functional hydrolysates if cost-effective technologies can be developed. Future efforts should focus on the development of products that satisfy the consumer's expectations for odor, color, taste, flavor, and appearance. Research should also focus more on other underrepresented fish species to shed light on potential applications in the field of protein hydrolysates from marine sources.

## Author contribution statement

All authors listed have significantly contributed to the development and the writing of this article.

## Funding statement

This work was supported by 10.13039/501100003725National Research Foundation of Korea grant (2022R1A5A8033794) under the Ministry of Science and ICT (MSIT), Regional Innovation Strategy (RIS) (2021RIS-002) under the Ministry of Education (MOE), and Cooperative Research Program for Agriculture Science and Technology Development (PJ01577005) under the Rural Development Administration, Republic of Korea.

## Data availability statement

Data included in article/supplementary material/referenced in article.

## Declaration of interest’s statement

The authors declare no conflict of interest.
